# Real-World Effect of a Digitally Delivered Conservative Musculoskeletal Care Program on Spinal Diagnostic Imaging Utilization in a Commercially Insured Population with Chronic Back Pain

**DOI:** 10.36469/001c.145231

**Published:** 2025-11-17

**Authors:** Louie Lu, Sandhya Yadav, Jeannie Bailey

**Affiliations:** 1 Clinical Research Hinge Health, Inc., San Francisco, California; 2 Department of Orthopaedic Surgery University of California, San Francisco

**Keywords:** digital health, imaging, back pain, conservative musculoskeletal care, medical claims

## Abstract

**Background:**

Conservative, noninvasive musculoskeletal treatment delivered digitally has demonstrated similar or better effectiveness in managing and reducing chronic back pain, compared to in-person care. However, there is limited evidence whether digital care reduces future spinal diagnostic imaging visits.

**Objectives:**

The primary goal was to examine the associations between participating in a digital conservative musculoskeletal care program for back pain and subsequent spinal diagnostic imaging use.

**Methods:**

Using medical claims data from a US commercial health plan database, this retrospective, secondary data analysis compared spinal diagnostic imaging visits among a group of digital program participants who had over 12 weeks of back pain to matched patients who only had usual care to treat their back pain. To mitigate selection bias, a propensity score matching model was developed to match study participants based on demographic, comorbidity, baseline medical care use and cost. The study outcomes were any spinal diagnostic imaging visit and number of spinal diagnostic imaging visits per 1000 participants up to 1 year after participating in the digital program.

**Results:**

The study included 2165 digital participants and 2165 matched comparison group patients. We found that digital participants had fewer spinal diagnostic imaging visits in the year after participating in the digital program compared with comparison group patients (14.2% vs 18.2%, *P* = .0003). The association between the digital program participation and spinal diagnostic imaging visit is stronger in the group who had imaging in the 12 months before, compared to those who had not (−4.8%, *P* = .007 vs −3.4%, *P* = .0163).

**Discussion:**

Consistent with previous studies demonstrating that early conservative management is associated with lower odds of imaging, findings from this study offer an encouraging direction for effective alternatives for managing back pain, improving performance outcomes and reducing premature utilization of healthcare services.

**Conclusion:**

The study provides evidence that participating in a digital musculoskeletal program that delivers conservative care is associated with fewer imaging use, especially among participants who had received imaging previously.

## INTRODUCTION

Chronic back pain, which encompasses a wide range of conditions affecting the diverse tissues of the spine (vertebrae, intervertebral discs, muscles, spinal cord and nerves, and connective tissues), frequently involves the use of diagnostic imaging techniques.^1–3^ When the patient presents appropriate symptoms, imaging offers many benefits as it can help providers to accurately diagnose the underlying conditions, effectively develop personalized treatment and care plans, and thus reduce the risk of unnecessary surgeries.^4^

However, imaging use for initial evaluation of low back pain has increased in the United States (US) since 2011.^5,6^ Recent studies have found that about one-third of imaging orders for lower back pain in the US were not prescribed with adherence to clinical guidelines.^7,8^ A systematic review of 33 studies by Jenkins et. al also found that 34.8% of patients referred for imaging had no symptoms for serious pathology or did not meet any criteria for clinical suspicion of pathology.^9^ Excessive or inappropriate use of these imaging techniques may lead to unintended consequences and potential harm.^6,10^ For example, magnetic resonance imaging (MRI) often detects in joints degenerative changes that may not be the cause of pain.^11^ It can result in more tests, specialist visits, and procedures or surgeries that may not be beneficial to the patient and that may increase healthcare costs.^12,13^ These surgeries may also result in infections, surgery complications, and prolonged recovery.^14^

Research has shown that conservative, noninvasive treatments such as physical therapy (PT), exercise, and patient education are effective in managing and reducing chronic back pain, improving function, and preventing future health care use including imaging.^15–21^ Influenced by the recent COVID pandemic, conservative care has been increasingly delivered digitally/virtually.^22^ Many digital musculoskeletal (MSK) programs take a biopsychosocial approach and provide education, health coaching, or cognitive behavioral therapy alongside exercise therapy to address chronic MSK pain. Recent studies have demonstrated superior clinical improvement in pain, functional status, and mental health among digital MSK participants compared with patients who received usual care.^23–27^

However, there is an absence of empirical evidence on how conservative MSK care delivered digitally may impact future imaging use compared with usual care (eg, office visit, in-person PT). The primary goal of this study was to examine the associations between participating in a digital conservative MSK program and subsequent spinal diagnostic imaging (SDI) use in the year after among digital MSK participants who had chronic back pain. We hypothesized that fewer patients would have an SDI after participating in the digital conservative care program. As a secondary goal of the study, we further examined whether any prior use of MSK-related imaging could affect subsequent SDI differently among digital participants and matched PT patients.

## METHODS

Using medical claims data from a US commercial health plan database, we conducted a retrospective secondary data analysis to compare digital MSK participants (“digital participants”) to propensity score–matched MSK patients who did not participate in the digital MSK program but received physical therapy (“PT patients”).

### Digital MSK Program Description

As described in a previous publication, the digital MSK program was an employment health benefit offered to employees and dependents aged 18 and over to help participants manage chronic MSK pain in back, knee, shoulder, hip and neck regions.^23^ The digital MSK program addressed chronic back pain by adopting personalized, behavioral guided services including exercise therapy, member education, and virtual visit with physical therapists and personal health coaches (**Supplemental Figure S1**). The program was accessed from an app on a participant’s tablet computer or smartphone. Each exercise therapy included 3 to 8 exercises that focused on stretching, strengthening, balance, and mobility. The participant was guided by animations and videos and had an option to receive feedback through using wearable motion sensors (InvenSense MPU-6050, TDK Electronics, Tokyo, Japan) regarding range of movement and repetitions. Participants also received educational resources after exercise and support from certified health coaches and PTs.

### Data Sources

We used Health Insurance Portability and Accountability Act (HIPAA)–compliant, de-identified medical claims data sourced from a US claims database that comprised more than 100 million commercially insured persons from January 1, 2016, through September 30, 2021, across all US states and territories. Data with enrollment dates and dates of service between January 2016 and September 2021 were included in this study.

### Data and Study Sample

The study included digital participants who enrolled in the chronic back pain pathway of the digital MSK program between January 2020 and October 2020 (“index event”). Digital participants may start the program by completing an exercise therapy, having a virtual PT visit, interacting with health coaches or accessing educational materials in the digital app. These participants were identified in the medical claims database using a validated privacy-preserving record linkage software by Datavant.^28–32^ To be HIPAA compliant, we were not able to link medical claims to pain score or engagement outcomes at the participant or group level (eg, number of exercises, coach interaction); therefore, this study took an intent-to-treat approach to examine the imaging outcomes among all engaged digital participants regardless of pain or engagement level.

In the same claims database, we selected a comparison group of individuals who did not enroll in the digital MSK program but had a back pain–related PT visit in the same month (“PT patients”) as the digital participants started the digital MSK program. Both groups had at least 1 chronic MSK related claim in the 12 months before the index event to indicate that they had a history of back pain. Therefore, the comparison group were nonparticipants who, at a minimum, had a PT evaluate their back pain and received in-person therapeutic exercises. Participants in either group could use other back pain–related care throughout the study period (eg, imaging). In addition, we excluded individuals who also had a back pain–related office visit in the same month as the index event, as they might be more likely to receive imaging in the near term than those who just started the digital program or traditional conservative care (eg, PT). For both groups, we included individuals who were 18 to 64 years old and continuously enrolled in a health plan at least 12 months before (ie, baseline period) and 12 months after (ie, post period) starting the digital MSK program or having their index event. Also, we excluded participants who had a surgery related to chronic MSK conditions before the index event, or had experienced cancer, pregnancy, or childbirth in the study period.

### Outcomes

The primary outcomes of this study were (1) percentage of study participants who had any SDI for chronic back pain and (2) number of SDI visits for chronic back pain during the index month, up to 3 months, 6 months, and 1 year since the index event. Usually, the digital participants had access to the digital program resources for a year and they could exercise and interact with the program on their own schedules. Therefore, we examined the imaging visits over these time periods (≤1 year). We also examined the specific modalities of spinal diagnostic imaging including standard x-ray and MRI. (We did not present the results on computed tomography (CT) in this study as the incidence of spinal CT scan is very low [0.8%].) We used the Current Procedural Terminology (CPT)/Healthcare Common Procedure Coding System (HCPCS) codes in the medical claims and the Restructured Berenson-Eggers Type of Service (BETOS) Classification System to identify the specific type of imaging.

### Statistical Methods

We applied a propensity score matching model to match the digital participants to the PT patients based on age group, gender, census division of residence, comorbidities, baseline chronic MSK and non-MSK medical spend quantiles, other concurrent MSK conditions, baseline MSK service use including injection, emergency room, PT, chiropractor, office visit (orthopedic surgeon and non-surgeon), imaging, lab test and use of any durable medical equipment in the 12 months before the index event. Comorbidities included hypertension, heart disease, diabetes, obesity, mental health needs, substance use disorders, autoimmune disorders, neurological disorders, and respiratory disorders. Specifically, we conducted matching separately for digital participants who had a prior MSK-related imaging vs those who had not. We matched the digital participants to the PT patients based on the calculated propensity score, using 1:1 nearest neighbor matching without replacement.

Many back pain patients may also have chronic MSK pain in other body regions such as the neck, knee, hip, and shoulder, which are also targeted by the digital MSK program. In our study sample, we identified 26% to 31% of back pain participants also had neck pain and 7% to 10% also had knee-, shoulder-, and hip-related pain in the 3 months prior to the index event. To better match PT patients with digital participants, especially for those with chronic MSK pain in multiple body regions, we included baseline chronic MSK services utilization for back, knee, shoulder, hip, and neck regions and also included a binary indicator for the 4 non-back regions if the individual had a clinical diagnosis in the medical claims in the 3 month prior to the index event including the index month.

For the primary and secondary outcomes, we generated descriptive statistics and conducted 2-sided *t* tests between the digital participants and matched PT patients. We reported means, 95% confidence intervals, mean differences, and *P* values. As a robustness check, we further conducted adjusted multivariable regression models that included all the matching covariates. Specifically, we applied a logistic regression for any SDI visit and a linear regression for the number of SDI visits. We also conducted a post hoc sample size calculation assuming power (0.80) and sample means (ie, any SDI within 1 year), the study needed 2640 to detect the difference in means between the digital participant and PT patient groups, given the sample standard deviation. The study (N = 4330) was sufficiently powered.

### Subgroup Analysis

We conducted an exploratory subgroup analysis on SDI visits among the prior imaging group vs the imaging-naive group. The prior imaging group differed from the imaging-naive group if a study participant had any diagnostic imaging on back, knee, shoulder, hip, or neck region in the 12 months prior to the index event. We compared the primary and secondary outcomes separately among the two groups and conducted *t* tests to detect any differences in SDI visits between the digital participants and the PT patients.

The study was reviewed and deemed exempt from institutional review board (IRB) oversight by WIRB-Copernicus Group® IRB (Office for Human Research Protections/FDA IRB registration number IRB00000533) at WIRB-Copernicus Group ® (1019 39th Avenue SE Suite 120, Puyallup, Washington 98374–2115).

## RESULTS

### Descriptive Findings

**Table 1** shows the demographics, comorbidity, baseline chronic MSK conditions and service utilization of the digital participants and PT patients before and after matching. Sample means, percentage point, volume difference, and *P* value were reported. After matching, 1002 digital participants who had a prior imaging in the baseline period were matched to 1002 of 97 570 PT patients who used imaging in the baseline period and had an index PT visit from January 2020 to October 2020 (“prior imaging group”). Among those who had not used any imaging in the baseline period, 1163 digital participants were matched to 1163 out of 145 174 PT patients (“imaging-naive group”). The final analytic sample included 2165 digital participants and 2165 matched PT patients.

**Table 1.** Baseline Characteristics Before and After Matching

	**Before Matching**				**After Matching**			
**Matched PT Patients**	**Digital Participants**	**Diff.**	***P* Value**	**Matched PT Patients**	**Digital Participants**	**Diff.**	***P* Value**	
Gender, %				<.001				.9030
Male	45.8	52.0			52.2	52.0		
Female	54.2	48.0			47.9	48.0		
Age group, %				<.001				.9530
18-29 y	10.6	2.0			2.1	2.0		
30-39 y	16.3	16.5			16.7	16.5		
40-49 y	25.6	26.6			25.8	26.6		
50-64 y	47.5	54.9			55.4	54.9		
Census division, %				<.001				.6020
New England	5.8	3.2			3.1	3.2		
Middle Atlantic	13.2	4.8			5.1	4.8		
East North Central	22.5	18.2			17.6	18.2		
West North Central	8.4	7.5			6.8	7.5		
South Atlantic	17.2	14.8			15.7	14.8		
East South Central	8.0	4.6			5.5	4.6		
West South Central	11.4	12.5			1.9	12.5		
Mountain	4.4	8.8			8.9	8.8		
Pacific	9.2	25.5			26.5	25.5		
Digital program participation start month/index month, %				<.001				.9930
Jan 2020	43.3	3.2			3.5	3.2		
Feb 2020	10.4	6.8			7.0	6.8		
Mar 2020	6.4	8.6			8.5	8.6		
Apr 2020	3.2	5.3			5.0	5.3		
May 2020	4.1	9.8			1.3	9.8		
Jun 2020	4.8	10.4			9.6	1.4		
Jul 2020	4.4	17.5			17.3	17.5		
Aug 2020	3.9	17.1			17.7	17.1		
Sep 2020	3.8	9.6			9.2	9.6		
Oct 2020	15.8	11.8			11.8	11.8		
Non-MSK cost quintiles, %				.022				.6050
1st	23.8	22.0			22.7	22.0		
2nd	22.3	20.5			2.9	2.5		
3rd	20.8	21.9			19.8	21.9		
4th	19.1	20.5			21.1	2.5		
5th	14.0	15.1			15.5	15.1		
MSK cost quintiles, %				<.001				.8380
1st	2.3	3.1			3.2	3.1		
2nd	35.3	40.7			39.2	4.7		
3rd	27.7	24.7			25.0	24.7		
4th	22.3	20.0			2.2	2.0		
5th	12.5	11.5			12.4	11.5		
Baseline comorbidity, %								
Cardiometabolic: hypertension	15.8	17.0	1.3	.1093	16.1	17.0	1.0	.3907
Cardiometabolic: heart	8.5	8.3	−0.2	.7117	8.4	8.3	−.1	.9126
Cardiometabolic: diabetes	7.2	8.1	1.0	.0891	8.3	8.1	−.1	.8681
Obesity	2.2	2.3	0.2	.6040	2.5	2.3	−.1	.7649
Mental health	18.1	17.9	−0.3	.7648	16.9	17.9	1.0	.3776
Substance use	1.3	1.4	0.1	.6124	1.6	1.4	−.2	.5322
Autoimmune	2.1	1.9	−0.1	.6748	1.7	1.9	.2	.5703
Neurological	8.0	8.1	0.1	.8141	7.2	8.1	.9	.2521
Respiratory	4.5	4.1	−0.4	.3495	4.2	4.1	−.1	.8790
Other concurrent MSK conditions, %								
Knee pain	10.2	8.4	−1.8	.0064	7.7	8.4	.7	.3712
Shoulder pain	9.1	8.5	−0.7	.2777	9.3	8.5	−.9	.3105
Hip pain	10.1	6.9	−3.2	<.0001	6.8	6.9	.1	.9521
Neck pain	31.2	25.9	−5.3	<.0001	27.3	25.9	−1.4	.3020
Baseline MSK service utilization per 1000, n								
Injections	390	500	110	.0062	470	500	30	.6537
ER visits	50	60	0	.5637	60	60	0	.9651
PT visits	16 600	10 100	−6490	<.0001	10700	10 100	−600	.3380
Chiropractor visits	7060	3780	−3290	<.0001	3850	3780	−70	.7168
MSK-related office visits	1320	1590	270	<.001	1750	1590	−160	.1918
Orthopedic surgeon office visits	160	220	60	<.001	240	220	−10	.5373
Imaging service in the baseline	1050	1140	90	.0190	1120	1140	20	.6787
DME-related service	100	80	−10	.6411	90	80	−10	.6978
N	242 744	2165			2165	2165		

Abbreviations: DME, durable medical equipment; ER, emergency room visit; MSK, musculoskeletal; PT, physical therapy.

In the matched sample, 52% of the study participants were male and 55% were in the 50- to 64-year age group. The most common comorbidities include hypertension, mental health, diabetes, and neurological disorders. One in four participants also had concurrent neck pain and 6% to 9% had pain in the knee, hip, or shoulder. The matched study participants on average had 4.7 to 4.9 PT visits, 0.7 to 0.8 MSK-related office visits, and 0.5 imaging visits in the 12 months before starting the digital program or index PT visit. After matching, the digital participants and PT patients did not exhibit any significant differences in demographics, comorbidities, baseline chronic MSK conditions and service utilization.

### Main Findings

**Table 2** illustrates the SDI use for digital participants vs the propensity score–matched PT patients. Sample means, 95% confidence internals, mean differences, and *P* values are reported. In the year after the index event, 18.2% (95% confidence interval [CI], 16.6%-19.8%) of the PT patients had at least l SDI visit, compared with 14.2% (95% CI, 12.7%-15.7%) of the digital participants (difference, 4.0%; *P* = .0003). Assuming the SDI use is 18.2% in the absence of the digital program, a 4 percentage-point difference means that 22% fewer digital participants had an SDI within 1 year after starting the digital program. In terms of imaging volume, digital participants had 90 fewer SDI visits per 1000 participants in the year after the index event, compared with the PT patients (*P* = .0001).

**Table 2.** Use of Spinal Diagnostic Imaging by Time Periods Since Index Event

	**Matched PT Patients**	**Lower Bound (95%)**	**Upper Bound (95%)**	**Digital Participants**	**Lower Bound (95%)**	**Upper Bound (95%)**	**Diff.**	***P* Value**
Participants who used any spinal diagnostic imaging, %								
Index mo	5.7	4.7	6.7	1.3	0.9	1.8	−4.3	<.0001
Up to 3 mo	10.0	8.8	11.3	4.1	3.2	4.9	−6.0	<.0001
Up to 6 mo	13.5	12.1	15.0	7.4	6.3	8.5	−6.1	<.0001
Up to 1 y	18.2	16.6	19.8	14.2	12.7	15.7	−4.0	.0003
Spinal diagnostic imaging per 1000 participants, n								
Index mo	61	50	72	15	9	21	−46	<.0001
Up to 3 mo	133	114	151	54	42	66	−79	<.0001
Up to 6 mo	206	181	231	104	86	122	−100	<.0001
Up to 1 y	315	281	350	226	198	254	−90	.0001
No. of observations	2165			2165				

Abbreviation: PT, physical therapy.

We also examined which imaging modality was mostly impacted by the digital program. We compared the imaging use outcomes for two of the most frequently used imaging services: standard x-ray and MRI. (We did not report other imaging modalities such as CT scan, as the numbers were very small.) Very few study participants in the matched sample used CT scan. Results are reported in **Table 3** by each period. Up to 1 year after the index event, 11.2% (95% CI, 9.9%-12.5%) of digital participants had a spinal x-ray compared with 13.8% (95% CI, 12.4%-15.3%) of matched PT patients. Also, fewer digital participants had a spinal MRI (6.1%; 95% CI, 5.1%-7.2%) vs PT patients (9.2%, 95% CI, 7.9%-10.4%). Compared with the PT patients, digital participants had 55 fewer (144 vs 199 per 1000 participants; *P* = .0008) spinal x-ray and 31 fewer (66 vs 97 per 1000 participants; *P* = .0005) spinal MRIs per 1000 participants within 1 year after starting the digital program.

**Table 3.** Use of Spinal Diagnostic Imaging by Imaging Modality

	**Matched PT Patients**	**Lower Bound (95%)**	**Upper Bound (95%)**	**Digital Participants**	**Lower Bound (95%)**	**Upper Bound (95%)**	**Diff.**	***P* Value**
Any spinal diagnostic x-ray, %								
Index mo	4.2	3.4	5.1	0.9	0.5	1.3	−3.3	<.0001
Up to 3 mo	7.4	6.3	8.5	2.9	2.2	3.6	−4.5	<.0001
Up to 6 mo	9.8	8.6	11.1	5.5	4.5	6.5	−4.3	<.0001
Up to 1 y	13.8	12.4	15.3	11.2	9.9	12.5	−2.6	.0088
Spinal x-ray per 1000 participants, n								
Index mo	42	34	51	9	5	13	−34	<.0001
Up to 3 mo	85	72	98	33	25	42	−52	<.0001
Up to 6 mo	127	109	144	65	53	78	−61	<.0001
Up to 1 y	199	174	224	144	124	164	−55	.0008
Any spinal diagnostic MRI, %								
Index mo	1.6	1.1	2.1	0.5	0.2	0.8	−1.1	.0003
Up to 3 mo	4.1	3.2	4.9	1.6	1.1	2.1	−2.5	<.0001
Up to 6 mo	6.6	5.5	7.6	2.8	2.1	3.5	−3.7	<.0001
Up to 1 y	9.2	7.9	10.4	6.1	5.1	7.2	−3.0	.0002
Spinal diagnostic MRI per 1000 participants, n								
Index mo	16	11	22	5	2	7	−12	.0002
Up to 3 mo	41	33	50	16	10	21	−25	<.0001
Up to 6 mo	67	57	78	29	22	36	−38	<.0001
Up to 1 y	97	84	111	66	55	77	−31	.0005
No. of observations	2165			2165				

Abbreviations: MRI, magnetic resonance imaging: PT, physical therapy.

As a robustness check, we conducted logistic and linear regression models to adjust for confounding factors that might not have been addressed in the propensity score model that could affect both groups. We included the full list of matching covariates to mitigate factors that might be associated with the study outcomes but not related to the digital program participation. **Table 4** presents results from unadjusted and adjusted regression models using the propensity score–matched analytical sample. Similar results were observed in the adjusted model that digital participants had significantly lower odds (odds ratio [OR], 0.73; 95% CI, 0.62-0.86) of having an SDI within a year of starting the digital MSK program vs PT patients. Digital participants on average had significantly fewer spinal imaging per 1000 participants (β, −95; 95% CI, −139 to −51) within 1 year compared with PT patients.

**Table 4.** Regression Model Results

	**Unadjusted**			**Adjusted**		
**OR or β**	**Lower Bound (95%)**	**Upper Bound (95%)**	**OR or β**	**Lower Bound (95%)**	**Upper Bound (95%)**	
Any spinal diagnostic imaging in a year	0.74^c^	0.63	0.87	0.73^c^	0.62	0.86
Any spinal diagnostic x-ray in a year	0.79^b^	0.66	0.94	0.78^b^	0.64	0.93
Any spinal diagnostic MRI in a year	0.65^c^	0.52	0.82	0.64^c^	0.51	0.81
No. of any spinal diagnostic imaging in a year per 1000 participants	−90^c^	−134	−45	−95^c^	−139	−51
No. of any spinal diagnostic x-ray in a year per 1000 participants	−55^c^	−86	−23	−58^c^	−90	−27
No. of any spinal diagnostic MRI in a year per 1000 participants	−31^c^	−49	−14	−32^c^	−50	−15

^a^*P* < .05.

^b^*P* < .01.

^c^*P* < .001.

### Subgroup Analysis

To examine whether the SDI use may differ between study participants who already had any imaging 12 months before their index event vs those who had not, we conducted a subgroup analysis to separately compare the imaging use outcomes within each group. **Table 5** shows the imaging use outcomes by these two groups. The differences in SDI use are larger among the prior imaging group. Among the prior imaging group, 4.8% fewer digital participants (17.5% vs 22.3%, *P* = .0072) have an SDI visit within 1 year after the index event, compared with 3.4% fewer digital participants in the imaging-naive groups (11.4% vs 14.7%, *P* = .0163). Consistent with the differences in imaging volume, digital participants had 120 fewer SDI visits per 1000 participants in the prior imaging group (284 vs 404, per 1000 participants; *P* = .0015), compared to 64 fewer SDI visits in the imaging-naive group (175 vs 239, per 1000 participants; *P* = .0194). The fewer imaging visits are mostly observed during the first six months since the index event.

**Table 5.** Use of Spinal Diagnostic Imaging by Prior Imaging Use

	**Matched PT Patients**	**Lower Bound (95%)**	**Upper Bound (95%)**	**Digital Participants**	**Lower Bound (95%)**	**Upper Bound (95%)**	**Diff.**	***P* Value**
**Prior imaging group**								
Participants who used any spinal diagnostic imaging, %								
Index mo	6.9	5.3	8.5	2.2	1.3	3.1	-4.7	<.0001
Up to 3 mo	13.0	10.9	15.1	5.7	4.3	7.1	-7.3	<.0001
Up to 6 mo	17.7	15.3	20.0	9.5	7.7	11.3	-8.2	<.0001
Up to 1 y	22.3	19.7	24.8	17.5	15.1	19.8	-4.8	.0072
Spinal diagnostic imaging per 1000 participants, n								
Index mo	76	58	94	25	14	36	-51	<.0001
Up to 3 mo	172	141	202	75	54	96	-97	<.0001
Up to 6 mo	270	229	312	135	105	164	−140	<.0001
Up to 1 y	404	346	462	284	239	330	−120	.0015
No. of observations	1002			1002				
**Imaging-naive group**								
Participants who used any spinal diagnostic imaging, %								
Index mo	4.6	3.4	5.9	0.6	0.2	1.1	−4.0	<.0001
Up to 3 mo	7.5	6.0	9.0	2.7	1.7	3.6	−4.8	<.0001
Up to 6 mo	10.0	8.3	11.7	5.7	4.3	7.0	−4.3	.0001
Up to 1 y	14.7	12.7	16.7	11.4	9.5	13.2	−3.4	.0163
Spinal diagnostic imaging per 1000 participants, n								
Index mo	48	35	61	7	2	12	−41	<.0001
Up to 3 mo	99	77	121	35	22	49	−64	<.0001
Up to 6 mo	150	121	180	77	56	98	−73	.0001
Up to 1 y	239	198	280	175	141	209	−64	.0194
No. of observations	1163			1163				

Abbreviation: PT, physical therapy.

## DISCUSSION

The primary goal of this study was to compare SDI use between digital participants and PT patients who were matched closely to the digital participants using a propensity score model that factored in demographic characteristics, comorbidities, baseline chronic MSK conditions and service utilization. The secondary goal was to explore if SDI use may be different if the study participants had a chronic MSK-related imaging visit in the 12 months prior to their index event. To our knowledge, this is the first study to examine the association of digital program participation and subsequent SDI utilization.

The study finds that participating in the digital program was associated with lower odds of having an SDI visit in the year after compared with the matched PT patients. Statistically significant differences in SDI use were observed in the first month up to 1 year after the index event. These results are consistent with existing literature that early conservative management (eg, PT) is associated with lower odds of imaging use.^20,21^ When comparing the differences by imaging modality, digital participants had both fewer spinal x-rays and MRIs.

Previous research in this area had focused on rates of compliance with diagnostic imaging guidelines in low back pain between PTs and primary care providers, and reported that PTs waited for a significantly longer time prior to the initial diagnostic imaging order.^10,33,34^ In this study, we theorized that as digital participants experience tangible improvements in pain and functional capacity through such holistic exercise programs; develop healthy behaviors through receiving effective health coaching, virtual PT and provider consultation; and become educated on their MSK conditions via the program’s member centric MSK educational resources, their inclination to get diagnostic imaging diminishes. Along with findings from previously studies on digital program engagement outcomes and clinical improvement, results in this study provide additional support that digital MSK programs may be a promising alternative to address low back pain and discourage SDI use.^35–39^

Diagnostic imaging use for back pain that is not adherent to the guideline is one of the few credible indicators of overuse in primary care.^40^ Several national clinical quality guidelines recommend that imaging should be limited to instances of progressive symptoms and clinicians not order imaging studies for nonspecific back pain.^5,41–43^ The National Committee for Quality Assurance’s Healthcare Effectiveness Data and Information Set (HEDIS) includes a measure, “Percentage of patients with a primary diagnosis of low back pain who did not have an imaging study (plain x-ray, MRI, or CT scan) within 28 days of diagnosis,” aiming to assess and promote appropriate use of imaging studies in the management of low back pain, with a focus on timely and evidence-based care.^40^ Despite the heightened awareness among providers and payers, the early adoption of imaging for low back pain has not decreased.^44,45^ There is a pressing need for effective strategies to curtail the utilization of low-value back pain–related imaging in primary care. The results of this study offer an encouraging direction for future interventions in MSK pain management, underscoring the importance of integrating digital health solutions as effective alternatives for managing low back pain, improving performance outcomes, and reducing premature utilization of healthcare services.

It is essential to interpret our findings while taking several limitations into account. First, as a claims based observational study, the study’s goal was to examine the association between digital MSK program participation and SDI use, instead of demonstrating any causal relationship. We constructed a closely matched comparison group using a quasi-experimental method and considered an extensive list of demographics and baseline MSK service use to mitigate the potential self-selection bias or confounding effects. Second, medical claims do not contain all the clinical data needed for this imaging study. The factors contributing to the early use of imaging in patients with low back pain likely involve a multitude of considerations outside what medical claims data can capture. For example, patients’ motivation on pursuing imaging, pain severity, and providers’ referral and practice patterns can potentially affect imaging use. It is also possible that some cases within our sample met the criteria outlined in the guidelines that allow for early imaging, such as instances of rapidly progressing neurologic loss. We could not rule out this possibility using de-identified medical claims. Similarly, we cannot be certain that only patients with initial occurrences of back pain were captured because not all pain can be captured in the medical claims. In other words, it is possible that individuals with a recurrent presentation of back pain, disguised as a “new” episode of back pain, might have been included in the study sample. These unobserved factors may ultimately influence the study estimates (ie, model misspecification) and should be examined in the future studies with additional data sources. Third, the group of individuals who participated in the digital program may be different from a typical patient with low back pain. Although we matched them to a group of similar PT patients, it is feasible that the digital participants are not representative to the back pain population. For example, the imaging use rate in both groups are lower than other studies.^3^

## CONCLUSION

We found that fewer digital participants had an SDI after participating in a digital program, and imaging avoidance was observed from the participation month and maintained within 1 year of the participation. These results suggest the digital MSK program may be a promising alternative in complying with clinical guidelines for use of diagnostic imaging for back pain.

### Disclosures

S.Y. and L.L. are employed by Hinge Health, Inc. S.Y. and L.L. have equity interest in Hinge Health, Inc.

### Ethics approval and consent to participate

The need for ethics approval and consent to participate was waived by Western Institutional Review Board (WIRB®), now known as WIRB-Copernicus Group (WCG® IRB) (OHRP/FDA IRB registration number IRB00000533). The study does not involve human participants and instead utilizes de-identified claims data for analysis. The data used in this retrospective study is de-identified, meaning that it does not contain any personally identifiable information and thus does not require consent from individuals.

## Supplementary Material

Online Supplementary Material
