# Supplementary material for: Real-World Effect of a Digitally Delivered Conservative Musculoskeletal Care Program on Spinal Diagnostic Imaging Utilization in a Commercially Insured Population with Chronic Back Pain
Source: J Health Econ Outcomes Res. 2025 Nov 17;12(2):200–8. doi: 10.36469/001c.145231 (PMC12629400; doi:10.36469/001c.145231)

Journal of Health Economics and Outcomes Research

Online Supplementary Material

Real-World Effect of a Digitally Delivered Conservative Musculoskeletal Care Program on Spinal Diagnostic Imaging Utilization in a Commercially Insured Population with Chronic Back Pain. *JHEOR*. 2025;12(2):200-208. doi:10.36469/jheor.2025.145231

ICD10 and CPT Codes Defining an Index Event for the Comparison Group

Table S1: CCSR Codes Defining Comorbidities

Table S2: Exclusion Criteria

Table S3: Full Logistic and Linear Regression Results Using Matched Sample

Figure S1: Digital MSK Program Description

This supplementary material has been provided by the authors to give readers additional information about their work.

This is an open-access article distributed under the terms of the Creative Commons Attribution 4.0 International License (CCBY-4.0). View this license's legal deed at http://creativecommons.org/licenses/by/4.0 and legal code at http://creativecommons.org/licenses/by/4.0/legalcode for more information.

ICD10 and CPT Codes Defining an Index Event for the Comparison Group

Back-Related ICD-10 Diagnosis Codes

G54.10, G54.40, G57.00, G57.01, G57.02, M12.18, M14.68, M14.88, M24.28, M25.78, M40.00, M40.05, M40.10, M40.15, M40.20, M40.205, M40.209, M40.295, M40.299, M40.30, M40.35, M40.36, M40.37, M40.40, M40.45, M40.46, M40.47, M40.50, M40.55, M40.56, M40.57, M41.00, M41.05, M41.06, M41.07, M41.08, M41.115, M41.116, M41.116, M41.117, M41.117, M41.119, M41.119, M41.125, M41.126, M41.127, M41.129, M41.20, M41.22, M41.25, M41.26, M41.27, M41.30, M41.35, M41.40, M41.45, M41.46, M41.47, M41.50, M41.55, M41.56, M41.57, M41.80, M41.85, M41.86, M41.87, M41.90, M42.00, M42.05, M42.06, M42.07, M42.08, M42.09, M42.10, M42.15, M42.16, M42.17, M42.18, M42.19, M42.9, M43.00, M43.05, M43.06, M43.07, M43.08, M43.09, M43.10, M43.15, M43.16, M43.17, M43.18, M43.19, M43.50, M43.5X5, M43.5X6, M43.5X7, M43.5X8, M43.5X9, M43.80, M43.8X5, M43.8X6, M43.8X7, M43.8X8, M43.9, M45.5, M45.5, M45.6, M45.6, M45.7, M45.7, M45.8, M45.8, M45.9, M45.9, M46.00, M46.05, M46.06, M46.07, M46.08, M46.09, M46.1, M46.40, M46.45, M46.46, M46.47, M46.48, M46.49, M46.80, M46.85, M46.86, M46.87, M46.88, M46.89, M46.90, M46.95, M46.96, M46.97, M46.98, M46.99, M47.015, M47.016, M47.019, M47.10, M47.15, M47.16, M47.20, M47.25, M47.26, M47.27, M47.28, M47.80, M47.81, M47.815, M47.816, M47.817, M47.818, M47.819, M47.89, M47.895, M47.896, M47.897, M47.898, M47.899, M47.9, M48.00, M48.05, M48.06, M48.07, M48.08, M48.10, M48.15, M48.16, M48.17, M48.18, M48.19, M48.20, M48.25, M48.26, M48.27, M48.40, M48.40XA, M48.40XD, M48.40XG, M48.40XS, M48.45, M48.45XA, M48.45XD, M48.45XG, M48.45XS, M48.46, M48.46XA, M48.46XD, M48.46XG, M48.46XS, M48.47, M48.47XA, M48.47XD, M48.47XG, M48.47XS, M48.48, M48.48XA, M48.48XD, M48.48XG, M48.48XS, M48.50, M48.50XA, M48.50XD, M48.50XG, M48.50XS, M48.55, M48.55XA, M48.55XD, M48.55XG, M48.55XS, M48.56, M48.56XA, M48.56XD, M48.56XG, M48.56XS, M48.57, M48.57XA, M48.57XD, M48.57XG, M48.57XS, M48.58, M48.58XA, M48.58XD, M48.58XG, M48.58XS, M49.80, M49.85, M49.86, M49.87, M49.88, M49.89, M51.05, M51.06, M51.06, M51.10, M51.15, M51.16, M51.16, M51.17, M51.17, M51.20, M51.25, M51.26, M51.26, M51.27, M51.27, M51.30, M51.35, M51.36, M51.36, M51.37, M51.37, M51.80, M51.85, M51.86, M51.86, M51.87, M51.87, M51.9, M53.2X7, M53.2X7, M53.2X8, M53.2X8, M53.3, M53.3, M53.80, M53.85, M53.86, M53.86, M53.87, M53.87, M53.88, M53.88, M53.9, M54.10, M54.15, M54.16, M54.16, M54.17, M54.17, M54.18, M54.18, M54.30, M54.30, M54.31, M54.31, M54.32, M54.32, M54.40, M54.40, M54.41, M54.41, M54.42, M54.42, M54.5, M54.80, M54.89, M54.9, M62.83, M99.03, M99.04, M99.13, M99.14, M99.23, M99.24, M99.33, M99.34, M99.43, M99.44, M99.53, M99.54, M99.63, M99.64, M99.73, M99.74, M99.83, M99.84,

Outpatient/Office Evaluation Codes

RBCS EV Category - CPT codes
98966, 98967, 98968, 99201, 99202, 99203, 99204, 99205, 99211, 99212, 99213, 99214, 99215, 99241, 99242, 99243, 99244, 99245, 99354, 99355, 99363, 99364, 99381, 99382, 99383, 99384, 99385, 99386, 99387, 99391, 99392, 99393, 99394, 99395, 99396, 99397, 99401, 99402, 99403, 99404, 99406, 99407, 99412, 99415, 99416, 99429, 99441, 99442, 99443, 99450, 99455, 99456, 99483, 99497, 99498, D0120, D0140, D0150, D0170, D0180, D0190, D4355, D9310, D9430, G0101, G0102, G0108, G0109, G0245, G0246, G0247, G0248, G0250, G0270, G0271, G0296, G0372, G0402, G0420, G0421, G0436, G0437, G0438, G0439, G0442, G0444, G0454, G0463, G0466, G0467, G0468, G0513, G0514, G0515, G9156, G9482,

G9483, G9484, G9485, G9486, G9487, G9488, G9489, G9873, G9874, G9875, G9876, G9877, G9878, M0064, Q0091

Physical Therapy Visit Codes

RBCS RT Category (exclude speech therapy) - CPT codes
97001, 97002, 97003, 97004, 97010, 97012, 97014, 97016, 97018, 97022, 97024, 97026, 97028, 97032, 97033, 97034, 97035, 97036, 97039, 97110, 97112, 97113, 97116, 97124, 97139, 97140, 97150, 97161, 97162, 97163, 97164, 97165, 97166, 97167, 97168, 97530, 97532, 97533, 97535, 97537, 97542, 97545, 97546, 97750, 97755, 97760, 97761, 97762, 97763, 97799, 97799, G0129, G0129, G0157, G0501

ICD10 diagnosis codes that define concurrent MSK conditions

Back-related ICD-10 Diagnosis Codes

Same as ICD10 Dx codes used in the index event service definition.

Hip related ICD-10 Diagnosis Codes

M12.051, M12.052, M12.059, M12.151, M12.152, M12.159, M12.251, M12.252, M12.259, M12.351, M12.352, M12.359, M12.451, M12.452, M12.459, M12.551, M12.552, M12.559, M12.851, M12.852, M12.859, M13.151, M13.152, M13.159, M13.851, M13.852, M13.859, M14.651, M14.652, M14.659, M14.851, M14.852, M14.859, M16.0, M16.10, M16.11, M16.12, M16.2, M16.30, M16.31, M16.32, M16.4, M16.50, M16.51, M16.52, M16.6, M16.7, M16.9, M21.051, M21.052, M21.059, M21.151, M21.152, M21.159, M21.251, M21.252, M21.259, M24.051, M24.052, M24.059, M24.151, M24.152, M24.159, M24.251, M24.252, M24.259, M24.351, M24.352, M24.359, M24.451, M24.452, M24.459, M24.551, M24.552, M24.559, M24.651, M24.652, M24.659, M24.7, M24.851, M24.852, M24.859, M25.051, M25.052, M25.059, M25.151, M25.152, M25.159, M25.251, M25.252, M25.259, M25.351, M25.352, M25.359, M25.451, M25.452, M25.459, M25.551, M25.552, M25.559, M25.651, M25.652, M25.659, M25.751, M25.752, M25.759, M25.851, M25.852, M25.859, M65.151, M65.152, M65.159, M66.151, M66.152, M66.159, M67.351, M67.352, M67.359, M67.451, M67.452, M67.459, M67.851, M67.852, M67.853, M67.854, M67.859, M70.60, M70.61, M70.62, M70.70, M70.71, M70.72, M71.051, M71.051, M71.052, M71.052, M71.059, M71.059, M71.151, M71.152, M71.159, M71.351, M71.352, M71.359, M71.451, M71.452, M71.459, M71.551, M71.552, M71.559, M71.851, M71.852, M71.859, M76.00, M76.01, M76.02, M76.10, M76.11, M76.12, M76.20, M76.21, M76.22, M95.5, M99.05, M99.15, M99.25, M99.35, M99.45, M99.55, M99.65, M99.75, M99.85

Knee-related ICD-10 Diagnosis Codes

M12.061, M12.062, M12.069, M12.161, M12.162, M12.169, M12.261, M12.262, M12.269, M12.361, M12.362, M12.369, M12.461, M12.462, M12.469, M12.561, M12.562, M12.569, M12.861, M12.862, M12.869, M13.161, M13.162, M13.169, M13.861, M13.862, M13.869, M14.661, M14.662, M14.669, M14.861, M14.862, M14.869, M17.0, M17.10, M17.11, M17.12, M17.2, M17.30, M17.31, M17.32, M17.4, M17.5, M17.9, M21.061, M21.062,

M21.069, M21.161, M21.162, M21.169, M21.261, M21.262, M21.269, M22.00, M22.01, M22.02, M22.10, M22.11, M22.12, M22.2X1, M22.2X2, M22.2X9, M22.3X1, M22.3X2, M22.3X9, M22.40, M22.41, M22.42, M22.8X1, M22.8X2, M22.8X9, M22.90, M22.91, M22.92, M23.00, M23.001, M23.002, M23.003, M23.004, M23.005, M23.006, M23.007, M23.009, M23.011, M23.012, M23.019, M23.021, M23.022, M23.029, M23.031, M23.032, M23.039, M23.041, M23.042, M23.049, M23.051, M23.052, M23.059, M23.061, M23.062, M23.069, M23.200, M23.201, M23.202, M23.203, M23.204, M23.205, M23.206, M23.207, M23.209, M23.211, M23.212, M23.219, M23.221, M23.222, M23.229, M23.231, M23.232, M23.239, M23.241, M23.242, M23.249, M23.251, M23.252, M23.259, M23.261, M23.262, M23.269, M23.300, M23.301, M23.302, M23.303, M23.304, M23.305, M23.306, M23.307, M23.309, M23.311, M23.312, M23.319, M23.321, M23.322, M23.329, M23.331, M23.332, M23.339, M23.341, M23.342, M23.349, M23.351, M23.352, M23.359, M23.361, M23.362, M23.369, M23.40, M23.41, M23.42, M23.50, M23.51, M23.52, M23.601, M23.602, M23.609, M23.611, M23.612, M23.619, M23.621, M23.622, M23.629, M23.631, M23.632, M23.639, M23.641, M23.642, M23.649, M23.671, M23.672, M23.679, M23.8X1, M23.8X2, M23.8X9, M23.90, M23.91, M23.92, M24.361, M24.362, M24.369, M24.461, M24.462, M24.469, M24.561, M24.562, M24.569, M24.661, M24.662, M24.669, M25.061, M25.062, M25.069, M25.161, M25.162, M25.169, M25.261, M25.262, M25.269, M25.361, M25.362, M25.369, M25.461, M25.462, M25.469, M25.561, M25.562, M25.569, M25.661, M25.662, M25.669, M25.761, M25.762, M25.769, M25.861, M25.862, M25.869, M65.161, M65.162, M65.169, M67.361, M67.362, M67.369, M67.461, M67.462, M67.469, M67.50, M67.51, M67.52, M67.861, M67.862, M67.863, M67.864, M67.869, M70.40, M70.41, M70.42, M70.50, M70.51, M70.52, M71.061, M71.061, M71.062, M71.062, M71.069, M71.069, M71.161, M71.162, M71.169, M71.20, M71.21, M71.22, M71.461, M71.462, M71.469, M71.561, M71.562, M71.569, M71.861, M71.862, M71.869, M76.50, M76.51, M76.52, M79.4

Shoulder related ICD-10 Diagnosis Codes

M12.011, M12.012, M12.019, M12.111, M12.112, M12.119, M12.211, M12.212, M12.219, M12.311, M12.312, M12.319, M12.411, M12.412, M12.419, M12.511, M12.512, M12.519, M12.811, M12.812, M12.819, M13.111, M13.112, M13.119, M13.811, M13.812, M13.819, M14.611, M14.612, M14.619, M14.811, M14.812, M14.819, M19.011, M19.012, M19.019, M19.111, M19.112, M19.119, M19.211, M19.212, M19.219, M21.211, M21.212, M21.219, M24.011, M24.012, M24.019, M24.111, M24.112, M24.119, M24.211, M24.212, M24.219, M24.311, M24.312, M24.319, M24.411, M24.412, M24.419, M24.511, M24.512, M24.519, M24.611, M24.612, M24.619, M24.811, M24.812, M24.819, M25.011, M25.012, M25.019, M25.111, M25.112, M25.119, M25.211, M25.212, M25.219, M25.311, M25.312, M25.319, M25.411, M25.412, M25.419, M25.511, M25.512, M25.519, M25.611, M25.612, M25.619, M25.711, M25.712, M25.719, M25.811, M25.812, M25.819, M61.011, M61.012, M61.019, M61.111, M61.112, M61.119, M61.211, M61.212, M61.219, M61.311, M61.312, M61.319, M61.411, M61.412, M61.419, M61.511, M61.512, M61.519, M62.011, M62.012, M62.019, M62.111, M62.112, M62.119, M62.211, M62.212, M62.219, M62.411, M62.412, M62.419, M62.511, M62.512, M62.519, M65.011, M65.011, M65.012, M65.012, M65.019, M65.019,

M65.111, M65.112, M65.119, M65.811, M65.812, M65.819, M66.111, M66.112, M66.119, M66.211, M66.212, M66.219, M66.811, M66.812, M66.819, M67.211, M67.212, M67.219, M67.311, M67.312, M67.319, M67.411, M67.412, M67.419, M67.811, M67.812, M67.813, M67.814, M67.819, M67.911, M67.912, M67.919, M70.811, M70.812, M70.819, M70.911, M70.912, M70.919, M71.011, M71.011, M71.012, M71.012, M71.019, M71.019, M71.111, M71.112, M71.119, M71.311, M71.312, M71.319, M71.811, M71.812, M71.819, M75.00, M75.01, M75.02, M75.10, M75.101, M75.102, M75.11, M75.111, M75.112, M75.12, M75.121, M75.122, M75.20, M75.21, M75.22, M75.30, M75.31, M75.32, M75.40, M75.41, M75.42, M75.50, M75.51, M75.52, M75.80, M75.81, M75.82, M75.90, M75.91, M75.92

Neck related ICD-10 Diagnosis Codes

G54.20, G54.30, M40.03, M40.04, M40.12, M40.13, M40.14, M40.202, M40.203, M40.204, M40.292, M40.293, M40.294, M41.02, M41.03, M41.04, M41.112, M41.113, M41.114, M41.122, M41.123, M41.124, M41.23, M41.24, M41.34, M41.41, M41.42, M41.43, M41.44, M41.52, M41.53, M41.54, M41.82, M41.83, M41.84, M42.01, M42.02, M42.03, M42.04, M42.11, M42.12, M42.13, M42.14, M43.01, M43.02, M43.03, M43.04, M43.11, M43.12, M43.13, M43.14, M43.3, M43.4, M43.5X2, M43.5X3, M43.5X4, M43.8X1, M43.8X2, M43.8X3, M43.8X4, M45.1, M45.1, M45.2, M45.2, M45.3, M45.3, M45.4, M45.4, M46.01, M46.02, M46.03, M46.04, M46.41, M46.42, M46.43, M46.44, M46.81, M46.82, M46.83, M46.84, M46.91, M46.92, M46.93, M46.94, M47.011, M47.012, M47.013, M47.014, M47.11, M47.12, M47.13, M47.14, M47.21, M47.22, M47.23, M47.24, M47.811, M47.812, M47.813, M47.814, M47.891, M47.892, M47.893, M47.894, M48.01, M48.02, M48.03, M48.04, M48.11, M48.12, M48.13, M48.14, M48.21, M48.22, M48.23, M48.24, M48.41, M48.41XA, M48.41XD, M48.41XG, M48.41XS, M48.42, M48.42XA, M48.42XD, M48.42XG, M48.42XS, M48.43, M48.43XA, M48.43XD, M48.43XG, M48.43XS, M48.44, M48.44XA, M48.44XD, M48.44XG, M48.44XS, M48.51, M48.51XA, M48.51XD, M48.51XG, M48.51XS, M48.52, M48.52XA, M48.52XD, M48.52XG, M48.52XS, M48.53, M48.53XA, M48.53XD, M48.53XG, M48.53XS, M48.54, M48.54XA, M48.54XD, M48.54XG, M48.54XS, M49.81, M49.82, M49.83, M49.84, M50.00, M50.01, M50.02, M50.03, M50.10, M50.11, M50.12, M50.13, M50.20, M50.21, M50.22, M50.23, M50.30, M50.31, M50.32, M50.33, M50.80, M50.81, M50.82, M50.83, M50.90, M50.91, M50.92, M50.93, M51.00, M51.04, M51.14, M51.24, M51.34, M51.84, M53.81, M53.82, M53.83, M53.84, M54.11, M54.12, M54.13, M54.14, M54.2, M54.6, M79.12, M95.3, M99.01, M99.02, M99.11, M99.12, M99.21, M99.22, M99.31, M99.32, M99.41, M99.42, M99.51, M99.52, M99.61, M99.62, M99.71, M99.72, M99.81, M99.82,

Table S1. CCSR Codes Defining Comorbidities

Comorbidity	Definition
Cardiometabolic: hypertension	A binary indicator identified by CCSR codes CIR007, CIR008
Cardiometabolic: heart	A binary indicator identified by CCSR codes CIR0011, CIR012, CIR019
Cardiometabolic: diabetes	A binary indicator identified by CCSR codes END002, END003
Obesity	A binary indicator identified by CCSR code END009
Mental health	A binary indicator identified by CCSR codes FAC007, MBD001-MBD013, SYM008
Substance use	A binary indicator identified by CCSR codes MBD017-MBD026, SYM009
Autoimmune	A binary indicator identified by CCSR diagnosis codes MUS003, MUS024, MUS025, MUS033, MUS034, MUS036
Neuro	A binary indicator identified by CCSR codes NVS004, NVS005, NVS010
Respiratory	A binary indicator identified by CCSR codes RSP008, RSP009

Table S2. Exclusion Criteria

Excluded Conditions	CCSR Diagnosis Codes
Cancer, neoplasms	NEO001-NEO025, NEO028-NEO71
Certain conditions originating in the perinatal period	Any PNL code
Pregnancy, childbirth, and the puerperium	Any PRG code

Table S3. Full Logistic and Linear Regression Results Using Matched Sample

	Any Back Imaging in a Year		Any Back X-ray in a Year		Any Back MRI in a Year		No. of Any Back Imaging in a Year per 1000 Participants		No. of Any Back X-ray in a Year per 1000 Participants		No. of Any Back MRI in a Year per 1000 Participants	
	**Unadjusted Model**	**Adjusted Model**	**Unadjusted Model**	**Adjusted Model**	**Unadjusted Model**	**Adjusted Model**	**Unadjusted Model**	**Adjusted Model**	**Unadjusted Model**	**Adjusted Model**	**Unadjusted Model**	**Adjusted Model**
Digital MSK program participation	0.743***	0.728***	0.785**	0.775**	0.650***	0.642***	-0.090***	-0.095***	-0.055***	-0.058***	-0.031***	-0.032***
	(0.062)	(0.062)	(0.073)	(0.073)	(0.076)	(0.076)	-0.023	-0.023	-0.016	-0.016	-0.009	-0.009
Female		1.070		1.072		1.000		0.001		0		-0.003
		(0.096)		(0.106)		(0.123)		-0.024		-0.017		-0.009
Age group												
30-39		1.112		0.953		0.723		-0.049		-0.001		-0.049
		(0.400)		(0.362)		(0.363)		-0.084		-0.06		-0.033
40-49		1.239		0.957		1.293		0.014		0.023		-0.014
		(0.435)		(0.354)		(0.620)		-0.082		-0.059		-0.033
50-64		1.425		1.176		1.335		0.041		0.045		-0.009
		(0.494)		(0.428)		(0.633)		-0.081		-0.058		-0.032
Census division												
Middle Atlantic		1.056		1.058		1.260		0.059		0.034		0.013
		(0.371)		(0.423)		(0.604)		-0.082		-0.058		-0.032
East North Central		1.814*		2.072*		1.340		0.144*		0.108*		0.025
		(0.539)		(0.699)		(0.564)		-0.07		-0.05		-0.028
West North Central		1.615		1.572		2.077		0.123		0.058		0.052
		(0.521)		(0.578)		(0.917)		-0.077		-0.055		-0.03
South Atlantic		1.782		1.687		1.728		0.11		0.059		0.039
		(0.533)		(0.576)		(0.723)		-0.071		-0.05		-0.028
East South Central		1.859		2.118*		1.285		0.118		0.081		0.026
		(0.618)		(0.789)		(0.619)		-0.082		-0.058		-0.032
West South Central		1.626		1.768		1.194		0.149*		0.113*		0.015
		(0.498)		(0.615)		(0.521)		-0.072		-0.052		-0.029
Mountain		1.228		0.958		1.758		0.053		0.004		0.039

		(0.392)		(0.357)		(0.765)		-0.075		-0.053		-0.029
Pacific		1.397		1.440		1.412		0.098		0.061		0.025
		(0.411)		(0.483)		(0.583)		-0.068		-0.049		-0.027
Digital program participation start month/index month (%)												
Feb2020		0.627		0.640		0.724		-0.067		-0.056		-0.007
		(0.179)		(0.206)		(0.304)		-0.076		-0.054		-0.03
Mar2020		0.657		0.619		0.925		-0.043		-0.035		0
		(0.183)		(0.195)		(0.369)		-0.074		-0.053		-0.029
Apr2020		0.719		0.848		0.881		-0.014		-0.003		-0.001
		(0.215)		(0.278)		(0.382)		-0.08		-0.057		-0.032
May2020		0.814		0.733		1.425		0.007		-0.015		0.029
		(0.215)		(0.219)		(0.532)		-0.072		-0.051		-0.029
Jun2020		0.899		0.835		1.457		0.024		-0.008		0.038
		(0.237)		(0.248)		(0.543)		-0.072		-0.052		-0.029
Jul2020		0.870		0.886		0.973		-0.01		0.005		0.001
		(0.217)		(0.247)		(0.354)		-0.068		-0.049		-0.027
Aug2020		0.998		0.946		1.204		0.038		0.034		0.014
		(0.246)		(0.262)		(0.430)		-0.068		-0.049		-0.027
Sep2020		0.983		1.073		1.397		0.065		0.045		0.026
		(0.258)		(0.313)		(0.523)		-0.073		-0.052		-0.029
Oct2020		0.821		0.856		1.063		0.013		0.021		0.008
		(0.212)		(0.248)		(0.397)		-0.071		-0.05		-0.028
Non-MSK Quintiles												
2nd Quntile		1.031		1.085		0.897		-0.002		0.006		-0.005
		(0.149)		(0.177)		(0.182)		-0.035		-0.025		-0.014
3rd Quntile		1.230		1.229		1.230		0.034		0.018		0.015
		(0.176)		(0.199)		(0.240)		-0.036		-0.026		-0.014
4th Quntile		1.467**		1.644**		1.233		0.083*		0.079**		0.012
		(0.210)		(0.262)		(0.244)		-0.038		-0.027		-0.015
5th Quntile		1.311		1.242		1.452		0.067		0.03		0.027
		(0.212)		(0.228)		(0.315)		-0.043		-0.031		-0.017

MSK Quintiles												
2nd Quntile		1.005		0.919		1.057		0.019		-0.001		0.008
		(0.279)		(0.278)		(0.410)		-0.068		-0.048		-0.027
3rd Quntile		1.025		0.967		0.947		0.002		-0.002		-0.004
		(0.292)		(0.301)		(0.379)		-0.07		-0.05		-0.028
4th Quntile		1.259		1.175		1.359		0.035		0.007		0.018
		(0.368)		(0.376)		(0.550)		-0.073		-0.052		-0.029
5th Quntile		1.424		1.262		1.452		0.061		0.018		0.029
		(0.464)		(0.454)		(0.645)		-0.086		-0.061		-0.034
Baseline MSK service utilization												
Number of injections		1.070**		1.067**		1.041		0.037***		0.031***		0.005
		(0.023)		(0.024)		(0.027)		-0.007		-0.005		-0.003
Number of ER visits		0.964		0.753		1.069		-0.03		-0.050*		0.012
		(0.107)		(0.114)		(0.145)		-0.034		-0.024		-0.013
Number of PT visits		0.997		0.999		0.996		-0.001		0		0
		(0.003)		(0.003)		(0.004)		-0.001		0		0
Number of chiropractor visits		0.988		0.992		0.975*		-0.003		-0.001		-0.002*
		(0.007)		(0.008)		(0.011)		-0.002		-0.001		-0.001
Number of MSK related office visits		1.006		0.992		1.020		0.001		-0.001		0.002
		(0.009)		(0.014)		(0.010)		-0.003		-0.002		-0.001
Number of orthopedic surgeon office visits		0.956		0.983		0.968		-0.014		-0.008		-0.003
		(0.058)		(0.066)		(0.078)		-0.018		-0.013		-0.007
Number of imaging service in the baseline		1.117***		1.103**		1.103**		0.044***		0.028***		0.011***
		(0.031)		(0.034)		(0.039)		-0.008		-0.006		-0.003
Number of DME related service		0.954		0.936		0.984		-0.012		-0.006		-0.003
		(0.059)		(0.069)		(0.074)		-0.016		-0.012		-0.006
Baseline Comorbidity												
Cardiometabolic: hypertension		1.146		1.175		1.048		0.064*		0.051*		0.005
		(0.129)		(0.145)		(0.164)		-0.032		-0.023		-0.013
		1.028		0.998		0.893		-0.022		-0.007		-0.012

Cardiometabolic: heart		(0.154)		(0.165)		(0.187)		-0.043		-0.03		-0.017
Cardiometabolic: diabetes		0.992		1.139		0.848		-0.038		-0.02		-0.016
		(0.150)		(0.185)		(0.184)		-0.042		-0.03		-0.017
Obesity		0.814		1.001		0.561		-0.075		-0.025		-0.044
		(0.227)		(0.294)		(0.244)		-0.075		-0.053		-0.03
Mental health		1.099		1.134		1.041		0.03		0.019		0.004
		(0.126)		(0.143)		(0.165)		-0.032		-0.023		-0.013
Substance use		0.910		1.100		0.928		0.066		0.073		-0.011
		(0.306)		(0.396)		(0.418)		-0.094		-0.067		-0.037
Autoimmune		1.820*		1.779*		1.270		0.109		0.064		0.03
		(0.480)		(0.507)		(0.475)		-0.085		-0.061		-0.034
Neurological		1.179		1.197		1.196		0.065		0.046		0.018
		(0.178)		(0.198)		(0.241)		-0.044		-0.031		-0.017
Respiratory		0.891		0.805		0.847		-0.033		-0.016		-0.017
		(0.186)		(0.191)		(0.250)		-0.057		-0.041		-0.023
Constant							0.315***	0.069	0.199***	0.024	0.097***	0.05
							-0.016	-0.137	-0.012	-0.098	-0.006	-0.054
R-squared							0.004	0.046	0.003	0.045	0.003	0.026
Adjusted R-squared							0.003	0.036	0.002	0.035	0.003	0.015
Observations	4330	4330	4330	4330	4330	4330	4330	4330	4330	4330	4330	4330

Note: * $p<0.01$; ** $p<0.001$; *** $p<0.0001$.

Figure S1. Digital MSK Program Description

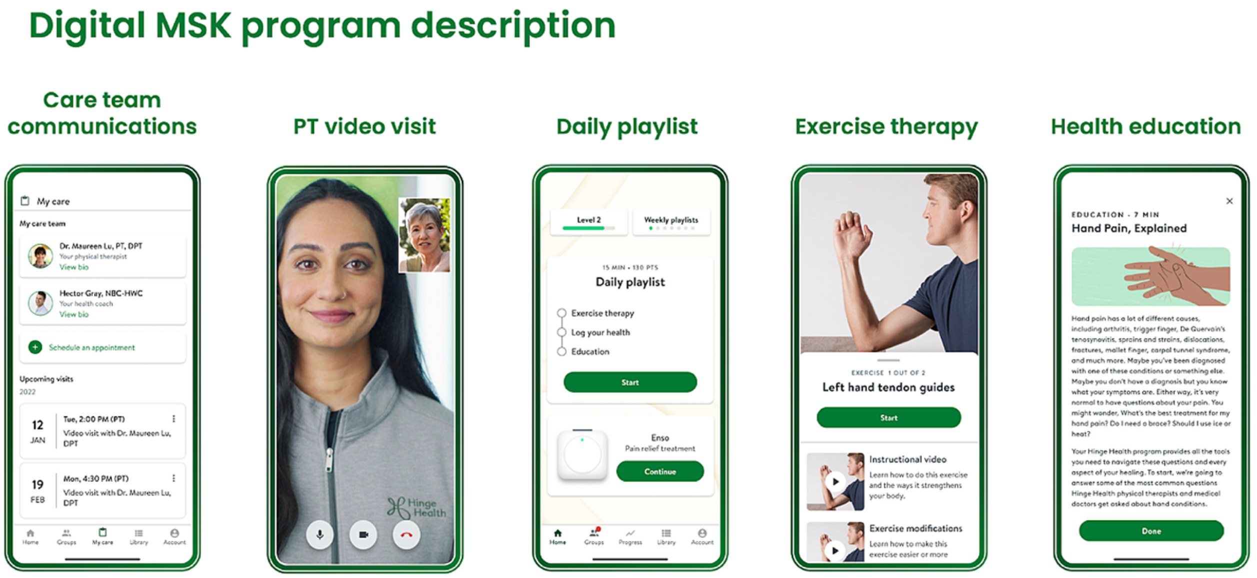

Supplement: Online Supplementary Material [file jheor_2025_12_2_145231_310831.pdf]
